# Supplementary material for: Acute otitis media symptoms and symptom scales in research with Aboriginal and Torres Strait Islander children
Source: PLoS One. 2023 Feb 23;18(2):e0280926. doi: 10.1371/journal.pone.0280926 (PMC9949645; doi:10.1371/journal.pone.0280926)
Supplement: Attachment — Submitted filename: Response to reviewers PONE-D-22-18129R1final.docx [file pone.0280926.s002.docx]

School of Medicine

Department of General Practice

Locked Bag 1797, Penrith NSW 2751

Building 30, Campbelltown Campus

Telephone: +61 414645062

14^th^ November 2022

Shengwen Calvin Li, PhD
Academic Editor
PLOS ONE

Dear Dr Li,

Re: PONE-D-22-18129 ‘Acute otitis media symptoms and symptom scales in research with Aboriginal and Torres Strait Islander children’

We thank the reviewers for their thoughtful comments and note the comments that the manuscript has merit, and that it presents a detailed and rigorous analysis of the use of two different scales – AOM-SOS and AOM-FS.

We have made the following changes to the manuscript.

**Academic Editor's Comments:**

**1) Response not required**

**2) All the Figures (1-4): Figure titles must carry self-explanatory information. An ideal figure title should give complete information to the reader even without reading the text. All the figure legend descriptions are not written in keeping this point in mind. All of these should be placed under the Figures; neither split part of the above and part of the below (e.g., Fig 3, Fig 4), nor all above the Figures**.

*We have augmented the Figure titles and legends as requested.*

**3) Lines 149 – 154: Brief descriptions should be provided in the figure legend, as resonated with the principle that an ideal figure legend should give complete information to the reader even without reading the text. E.g., * shaded items correspond to the AOM-SOS version 3.0 – what was the version?**

*Please see above*

**4) Besides what was presented in the manuscript, are there other demographic characteristics (e.g., laboratory results that might contribute to the syndromes and imaging upper respiratory tract infections)? A few specific cases (individual patients) will be helpful to illustrate the complexity of the disease. Some outcome measurements might be relevant.**

*We agree that this information would contribute to our paper, but this was beyond the scope of our data collection and we have no laboratory or other results to determine infectious aetiology. WATCH was designed as a pragmatic trial of current management practices for AOM and we used existing AOM symptom scales developed for research. We argue that our findings in this paper - which suggest limited usefulness of these scales in our population of Aboriginal and Torres Strait children - will be useful to other researchers and may promote more research of the type you suggest.*

*We can provide model case vignettes if needed by the journal but believe this will not contribute well given the variety (and vagueness) of diagnostic criteria used by clinicians and guidelines for AOM. We believe our clarifications in this paper (see point 6) will assist the reader in the way you have identified is needed.*

**5) Lines 111 – 113: "Figure 1 Acute Otitis Media – Faces Scale" the circular scheme of the current version is confusing. One dimension of the schematic diagram might better illustrate escalated scaling, which should be more concrete in the frequent description.**

*We have presented the version as used in our study and as previously published and validated in a US population (ref 9). In our view it is important to present it exactly as used, so readers can assess its strengths or limitations for the parent/carers in our study.*

**6) Lines 411-420: A schematic diagram showing the different criteria for an AOM diagnosis between the international standard and the method used in the manuscript should be provided to govern the current study. Ideally, a measurement matrix derived from the study should be proposed, i.e., a diagnostic flow chart for both AOM-SOS and AOM-FS symptom scales.**

*Thank you for the careful reading of this paper and identification of the lack of clarity related to diagnosis of AOM which made our paper harder to absorb. We have reviewed the whole paper for where we may not have been clear.*

- *We argue there is no one international standard for diagnosis of AOM. We have prepared the following table which could be included in supplementary materials comparing the Australian and the US guidelines. However, we do not feel this table will add substantially to our paper. This is a debated area and most national guidelines do not define diagnostic criteria for AOM in their AOM management guidelines. (for the diversity of European AOM guidelines and the evident focus on management without definition of diagnosis see reference Suzuki et al 2020 Clinical practice guidelines for acute otitis media in children: a systematic review and appraisal of European national guidelines. 2020 May 5;10(5):e035343.)*
- *To increase research reproducibility, researchers are clear in their diagnostic criteria in their studies, and they often differ. We also have been very clear on our definition of AOM in our study and acknowledge the effect this has had on our research and use of symptom scales. However, we believe that because we did not include symptoms other than pain (as alternate or addition to the sign of tympanic membrane bulge) to recruit patients to our study we are in a strong position to examine symptoms of AOM. This will be of interest to AOM researchers.*

*To clarify diagnostic issues in AOM and OM relevant to this paper (prior to the detailed pre-existing discussion of the issue starting line 425) we have made these changes:*

*BACKGROUND:*

- *Clarified that we have used the most stringent criteria for AOM diagnostic accuracy according to the Australian guidelines (line 63): ‘Acute OM (AOM) is a middle ear infection most reliably diagnosed by middle ear effusion associated with bulging of the tympanic membrane, and/or ear pain or recent discharge of pus according to Australian guidelines.’*
- *Updated the Australian guideline on which our criteria are based to new version (ref 1) This also led to adjustment in Box 1.*
- *Clarified AOM and subsequent chronic OM: line 67- The natural history of AOM is to resolve spontaneously within 1-2 weeks, although it may persist and lead to chronic OM and conductive hearing loss and other adverse health outcomes’ and line 75 - added the word chronic.*

*METHODS*

- *Increase clarity about OM and perforations line 142 such as a current or past history of tympanic membrane perforation,*
- *clarified that AOM-SOS and AOM-FS findings were not part of the enrolment decision making (143): “The AOM (SOS) and AOM- FS were not included in the determination of eligibility for enrolment.”*

*DISCUSSION*

- *reemphasised the stringent diagnostic criteria point line 422: regardless of whether children’s AOM had resolved by day 7 or not according to stringent AOM diagnostic criteria*

*ABSTRACT:*

- *Better worded how we used the scales in the trial associated with this paper – line 34 abstract “Two scales were employed to monitor symptoms over time…”*
- *Reinforced the point about stringent diagnostic criteria line 58: ‘However, it is likely the scales measured concurrent symptoms related to upper respiratory tract infections given they did not differentiate children with persistent or resolved AOM based on stringent diagnostic criteria.’*

|  | *Otitis Media Guidelines for Aboriginal and Torres Strait Islander Children. Australian Government Department of Health and Ageing 2017*  ***(our study)*** | *American Academy of Pediatrics Guideline 2013 (two scenarios given)* | |
| --- | --- | --- | --- |
| Evidence of middle ear effusions (MEE) | MEE on tympanometry (i.e., Type B) | MEE on tympanometry or Pneumatic otoscopy | MEE on tympanometry or Pneumatic otoscopy |
|  | AND | AND | AND |
| Bulge | Any bulge | Moderate to severe bulge | Mild bulge |
|  | AND/OR | OR | AND |
| Other symptoms and signs | Ear pain* | New onset otorrhoea  (not due to acute otitis externa) | Recent (<48 hrs) onset ear pain (holding tugging, rubbing of the ear in a nonverbal child) or intense erythema of the TM |

** Irritability was sufficient for ear pain among infants <3 years
AOM = Acute otitis media*

*TM = Tympanic membrane*

**7) Conclusions: Write brief statements on the results obtained per the study's objectives.**

*We have added sentences as underlined to the conclusion to very clearly link to the two aims of our study as expressed at the end of the Background:*

*Our research provides new information on AOM symptoms in urban Aboriginal and Torres Strait Islander children. Minimally symptomatic AOM appears to be common in Aboriginal and Torres Strait Islander children living in urban communities. Given their high burden of ear and hearing problems, routine and opportunistic ear screening may be necessary to adequately detect AOM.*

*Our examination of the function and usefulness of the AOM-FS and AOM-SOS scales as research tools in the WATCH trial leads us to question the value of these scales in our study population. Despite functioning similarly, the AOM-SOS and AOM-FS symptom scales did not discriminate between children with resolved and persistent AOM and it appears likely the scales were largely measuring symptoms related to concurrent upper respiratory tract infection. . The value of the scales as research or clinical tools among Aboriginal and Torres Strait Islander children, a population at high risk of otitis media and its complications, appears limited.*

**Reviewer #1:**

**1. No real figure legend is provided. “Note” is added at the end of Figures 3 and 4. But a real figure legend is needed.**

*Please see above*

**2. In Figure 4, some days show weird string-like shape. It seems that the values are either discrete integers, or close to integers. Why is this? Any such trend is not shown in other days? Full explanation is needed.**

*The Figure legends for Fig 3 and 4 have been amended to include the statement that the width of the figure represents the number of results with that score. We have also included boxplots and actual data values in the graphs, to show the full data distribution of scores for each time point (the latter jittered to showcase visibility of the data within each score point, which is a discrete value). We believe the visual representation will now provide readers with better understanding of our data, thank you.*

**3. Figure 4. Better visualization is needed. I do not think violin plot is the good choice here. Better label is also needed here.**

*We have created new graphs to show the distribution of scores over time. We have used a combination of a violin, boxplot, and actual data values to show AOM scores variability and it’s change over time. We have amended the Figure titles and legends, and hope this clarifies the Figure and better explains the results.*

**4. Table 2. Second line from last, right column, the authors claim that the relative change from base line is 100%. But this is incorrect. The relative change is in fact infinity (or does not exist) since it is equal to 1/0.**

*Thanks, we have changed to “Not applicable”*

**5. Page 12, line 218. What is “rs”. This is not standard notation. I suspect that authors want to use Pearson correlation “r” but I am not sure. Please double check and provide explanation. What are the p-values for day 3 and day 7?**

*We use the notation r_s_ to denote Spearman’s correlation (as per APA style), to distinguish between the usual Pearson’s correlation which is denoted by r. We have now added p-values for days 3 and 7.*

**Reviewer #2: The manuscript presents a detailed and rigorous analysis of the use of two different scales – AOM-SOS and AOM-FS – to monitor AOM in diagnosed patients. The strength of the manuscript is the critical analysis of the limitations of the study, namely, low symptom severity at enrollment, difficulty distinguishing between symptoms related to AOM and upper respiratory tract infections, as well as the limitations of each scale system. I only have a few minor suggestions and requests:**

**1. Label figure panels with letters and refer to them accordingly in the captions and the text.**

*Thank you, amended as noted above*

**2. State the statistical significance of the decrease in AOM-SOS and AOM-FS scores over time (Figs. 3-4 and corresponding sections in the main text).**

*Thank you for the suggestion, we have now included significance testing results.*

**3. I recommend splitting the second sentence of the abstract into two sentences: “We collected data on symptoms associated with acute otitis media (AOM) in a clinical trial involving children receiving primary care at urban Aboriginal Medical Services. Two scales were employed to monitor symptoms over time: the AOM-Severity of Symptoms scale (AOM-SOS) and the AOM-Faces Scale (AOM-FS)”.**

*Thank you for the suggestion, we have made this amendment.*

Kind regards,

Professor Penelope Abbott
